# Clustering of lifestyle risk factors among adult population in India: A cross-sectional analysis from 2005 to 2016

**DOI:** 10.1371/journal.pone.0244559

**Published:** 2021-01-04

**Authors:** Rufi Shaikh, Junaid Khan

**Affiliations:** 1 Sankhya Analytical Research Pvt.Ltd, Mumbai, India; 2 International Institute for Population Sciences (IIPS), Mumbai, India; Institute of Economic Growth, INDIA

## Abstract

**Introduction:**

Individual’s early life style and health behaviors are directly linked to chronic non-communicable diseases. Considering the increased burden of NCDs during the last two decades, the aim of this study is to assess co-occurrence/clustering of lifestyle risk factors and its association with different socio-demographic and economic characteristics among adult men and women in India from 2005–2016.

**Methods:**

This study utilized the data from the National Family Health Survey 2005–06 and 2015–16 survey rounds. Multinomial logistic regression is employed to evaluate co-occurrence of multiple risk factors among adult men and women of different socio-economic and demographic characteristics to identify the subgroups with elevated risk of clustering of multiple unhealthy lifestyle risk factors.

**Results:**

More adult men in India tend to exhibit clustering of multiple non-communicable disease risk factors than females. Individuals between 30–49 years of age, residing in urban areas, the population with no education, separated couples and those from poor economic strata are the specific population subgroups show higher prevalence of co-occurrence of multiple risk factors. The regional pattern of clustering of risk factors shows that the prevalence of co-occurrence of multiple risk factors is higher among men and women from the North-Eastern part of India compared to the other regions of the country.

**Conclusion:**

The prevalence of clustering of multiple risk factors associated with chronic NCDs is substantially high and has increased between 2005–06 to 2015–16. India may therefore experience a significant increase in the burden of chronic non-communicable diseases in the coming years. We therefore conclude that appropriate strategies should be implemented by policy makers and the government to reduce the overall health burden of NCDs due to lifestyle habits.

## Introduction

Life expectancy in India is increasing, causing a shift from Group I (communicable) diseases to Group II (chronic and non-communicable) diseases [1]. Chronic non-communicable diseases (NCDs) account for approximately 60% of total mortality in the world, with around 80% of these deaths occurring in developing countries [2,3]. India is the second most populous country in the world where 55% of the disability adjusted life years (DALY’s) in 2016 were attributed to chronic non-communicable diseases [4]. The Global Burden of Disease Study data shows that dietary habits, tobacco and alcohol use, blood sugar, obesity and low physical activities are major metabolic and behavioural risk factors which lead to non-communicable diseases in India [4]. Life-style behaviours are the most crucial determinant [5,6] and lie at the root of many chronic diseases [7–14] causing higher disease burden [15] and premature mortality worldwide [16,17]. There is evidence that lifestyle risk factors like tobacco smoking and chewing, alcohol consumption, unhealthy diet are associated and tend to co-occur [8,18,19] as clusters [9,10,20] or in bundles [6]. Evidence suggests that population that indulge in multiple health risk factors tend to have significantly worse health outcomes than those engaging in one health risk behaviour [15,21].

Linkages between lifestyle behaviours and socio-demographic characteristics had been studied previously mostly by analysing individual’s lifestyle health risk behaviours [22–26]. The only study for India which estimated the association between aggregation of health risk factors was based only on six selected states (Assam, Karnataka, Maharashtra, Rajasthan, Uttar Pradesh and West Bengal) in the country and the results were generalized for India as a whole [27]. As India constitutes of 29 states and 7 union territories, co-occurrence of multiple lifestyle risk factors may predispose the population to a higher burden of NCDs which are more detrimental to health compared to single lifestyle risk factors. Additionally, the identification of population subgroups with co-occurrence of multiple risk factors might facilitate the development of specific health promotion strategies for those vulnerable population groups. In particular, the systematic analysis of clustering of major risk factors of NCDs is lacking to claim national representation for Indians. Therefore, the objective of this paper is to investigate the degree of co-occurrence/clustering of lifestyle risk factors among adult Indian population of different socio-demographic characteristics over time and to identify the subgroups with clustering of multiple risk factors.

## Material and methods

### Data source

We use data from the third and fourth round (2005–06 and 2015–16) of the National Family Health Survey (NFHS) to estimate national and regional level prevalence of clustering of unhealthy lifestyle risk factors. The NFHS is a nationally representative, cross-sectional, household based sample survey representing 99 percent of the Indian population living in 29 states and 7 union territories [28,29]. It is one of the most important population surveys in India which provides reliable estimates for various socio-demographic indicators, child nutrition and mortality, reproductive health, morbidity, health services and other aspects of health including lifestyle habits and sexual behaviour for the population aged 15–49 for females and 15–54 for males. The sample size (109,041 households in 2005–06 & 601,509 households in 2015–16) is large enough to provide reliable estimates for the country and its states for both the rounds of survey. A sample of 78,586 women aged 15–49 and 41,847 men aged 15–49 from NFHS, 2005–06 and another sample of 671,626 women aged 15–49 and 101,611 men aged 15–49 from NFHS, 2015–16 constitute the analytical sample of this study.

### Statistical analysis

Bivariate cross tabulation analysis is performed to estimate the sex specific prevalence of clustering or co-occurrence of unhealthy lifestyle behaviours such as smoking, chewing tobacco, alcohol consumption, unhealthy food habits, obesity and hypertension by socio-economic and demographic characteristics. Sex specific two different multinomial logistic regression analysis are employed to evaluate the likelihood of co-occurrence of multiple risk factors among adult men and women of different population characteristics. No or one risk factor is considered to be the reference category for the analysis. Due to low frequency of simultaneous presence of all six risk factors during 2015–16, presence of five and six non-communicable disease risk factors are combined together. We adopt 95 percent, 99.5 percent and 99.9 percent as the confidence limits for all statistical tests. The statistical analyses are carried out using Stata®15.1 version.

#### Dependent variables

Lifestyle risk factors are ascertained as part of a self-administered questionnaire in the NFHS. To define obesity and hypertension, we use the cut-off points based on the World Health Organization (WHO) guidelines. As per the WHO guideline, the cut-off point for obesity in terms of body mass index (BMI) is 30kg/m^2^, which is associated with morbidity and mortality. Similarly WHO also recommends a cut-off of systolic blood pressure reading as ≥140 mmHg and/or the diastolic blood pressure reading as ≥90 mmHg as hypertensive. Body mass index is used as a proxy measure of physical inactivity and sedentary behaviour among the population. Different types of smoking like cigarette smoking, use of “Bidis” and “Hookah” are combined together. Unhealthy food intake prevalence is estimated on the basis of never consumed and daily/occasionally consumed food habits of individuals. NFHS does not provide the specific information on the number or frequency of smoking, alcohol consumption, chewing tobacco and quantity of food consumed which is an important data limitation of the study. Clustering of risk factors is identified by the simultaneous occurrence of one or more risk factors.

#### Outcome variable

To classify the individuals, a score ranging from 0 to 6 is used where ‘0’ represents no risk factor, ‘1’ represents presence of only one risk factor, ‘2’ represents simultaneous presence of two risk factors, ‘3’ represents simultaneous presence of three risk factors. Likewise, a score of ‘6’ represents simultaneous presence of six risk factors. The 2015–16 survey includes hypertension as one additional risk factor which is not present in the 2005–06 survey round of NFHS. The group with one or no risk factor is considered as the reference category in the multivariate framework.

Description of lifestyle risk factors used in the present study is as follows:

**Table:** 

NCD Risk Factor	Description
Smoking	Does he/she smoke tobacco or other related products?
Alcohol	Does he/she drink alcohol?
Smokeless Tobacco	Does he/she chew or use tobacco or other related products?
Healthy Food Habit	Does he/she consume milk or other products/vegetables/fruits/eggs/fish/meat daily/weekly/occasionally/never?
Obesity	WHO standard for BMI greater than or equal to 30kg/m^2^.
Hypertension*	WHO standard for Systolic blood pressure greater than or equal to 140mmHg and Diastolic blood pressure greater than or equal to 90mmHg.

* The 2015–16 round of data includes hypertension as one more subject as a risk factor which is not present in 2005–06 data.

#### Independent variables

Data on age, place of residence, education, living arrangement, Indian regions and economic status are obtained from the dataset. As the study sample is restricted to age 15–49 for both men and women, the age variable is dichotomized into two categories, 15–29 years old and 30–49 years old to ensure homogenous distribution of frequency in each category. Educational attainment is categorized into illiterate, primary, secondary and higher education, place of residence is dichotomized into urban and rural, living arrangement is classified as never married, living with partner and not living with partner and economic status is classified into poor, middle and rich based upon the wealth quintile variable. The lowest two wealth quintiles- poorest and poorer are defined as poor (in general) and the two upper wealth quintiles-richer and richest are considered to be rich in this study. As per the definition of NFHS, the six regions of India are North, East, North-East, West, Central and South.

## Results

Table 1 shows the descriptive statistics of the study population. Of the total respondents from 2005–06 survey round, 45 percent are men and 47.4 percent are women who belong to age 15–29 years whereas 55 percent of the men and 52.6 percent of the women belong to age 30–49 years. Similarly, there are 49.8 percent and 50.2 percent men aged 15–29 and 30–49 years and 49.3 percent and 50.7 percent women belong to age 15–29 years and 30–49 years respectively. Of the total surveyed population from 2005–06 NFHS, 16.3 percent men and 28.1 percent women have no formal education whereas only 17.9 percent men and 12.2 percent women from the 2015–16 NFHS are found to have higher educated. The economic status of the population has declined during the ten year of 2005–2016 with increased proportion of the population living in the poor economic quintile in 2015–16 as compared to 2005–06.

**Table 1:** Characteristics of study population, India, NFHS, 2005–06 & 2015–16.

Socio-demographic and Economic Variables	2005–06				2015–16			
	Men		Women		Men		Women	
	N = 41,847		N = 78,586		N = 101,611		N = 671,626	
Age	Percentage	Total	Percentage	Total	Percentage	Total	Percentage	Total
15–29	45.01	19,455	47.44	39,027	49.82	50,982	49.30	3,49,518
30–49	54.99	22,392	52.56	39,559	50.18	50,629	50.70	3,22,108
**Residence**								
Urban	39.26	22,331	50.88	38,298	38.25	32,201	30.95	1,94,940
Rural	60.74	19,516	49.12	40,288	61.75	69,410	69.05	4,76,686
**Education**								
No education	16.31	5,252	28.06	23,097	11.80	12,324	27.30	1,88,891
Primary	15.87	5,995	14.07	11,285	11.95	12,379	12.48	84,417
Secondary	52.12	22,845	45.04	34,899	58.34	60,615	48.05	3,21,731
Higher	15.70	7,755	12.82	9,305	17.91	16,293	12.18	76,587
**Living Arrangement**								
Never married	29.26	14,178	19.49	16,185	27.20	20,828	22.28	1,62,403
Living with partner	68.93	27,049	75.46	58,408	65.02	50,480	73.41	4,80,559
Not living partner	1.81	620	5.05	3,993	7.78	6,169	4.32	28,664
**Economic level**								
Poor	28.81	8,199	18.51	16,116	33.44	37,767	37.15	2,71,094
Middle	19.80	7,904	17.04	14,137	21.29	22,145	20.95	1,41,411
Rich	51.39	25,744	64.45	48,333	45.27	41,699	41.90	2,59,121
**Indian Region**								
North India	28.83	11,569	29.92	23,129	27.90	35,520	35.33	2,32,273
Central India	8.17	2,457	7.80	6,316	8.42	12,925	12.29	85,870
East India	20.46	3,706	13.13	10,748	18.64	15,570	16.74	1,19,820
North East India	3.87	6,209	16.42	13,460	3.25	12,592	13.50	90,350
South India	22.84	11,579	19.98	15,093	23.35	13,812	14.05	89,002
West India	15.82	6,327	12.74	9,840	18.45	11,192	8.09	54,311

Table 2 presents the co-occurrence of lifestyle risk factors in India by gender. It can be seen that clustering of unfavourable risk behaviours is higher among men as compared to women. Approximately 36 percent men show simultaneous presence of at least three health risk behaviours during both the time point. Prevalence of co-occurrence of at least three lifestyle risk factors among women increased from 8.98 percent during 2005–06 to 10 percent during 2015–16.

**Table 2:** Co-occurrence of lifestyle risk factors among adults in India.

No of Lifestyle Risk Factors	Percentage (%)			
	2005–06		2015–16	
	Men	Women	Men	Women
Zero or one	36.00	69.43	36.60	65.24
Two	22.84	21.59	27.76	24.76
More than three	41.16	8.98	35.64	10.01

Table 3A and 3B gives the percentages of simultaneous presence of chronic risk factors across gender by different socio-demographic and economic characteristics during 2005–06 and 2015–16. Among both men and women, clustering of 2 or more unfavourable lifestyle risk factors has increased between 2005–06 and 2015–16. From the table, it is observed that 13 percent of men and 20 percent of women during 2005–06 do not show any presence of risky lifestyle behaviour leading to chronic diseases and the prevalence show a drop to 8.63 percent for men and 18.76 percent for women during 2015–16. Apparently, this indicates that the prevalence of clustering of at least one risk factor has increased among Indian adult population. The simultaneous presence of two or more risk factors is higher among individuals who belong to age group 30–49 years, population with no education and among divorced or separated. It is also observed that the sub-population from the poorest economic stratum carry the highest prevalence of simultaneous occurrence of three or more risk factors during both the time points. Notably, the richest wealth quintile carry the highest burden of simultaneous occurrence five or more risk factors during 2005–06. Also, when we group the country into its six different regions based on the demographical structure, it is observed that men and women residing in the North-Eastern part of the country have a proliferated presence of multiple lifestyle risk factors.

**Table 3:** **a. Prevalence of co-occurrence of non-communicable disease risk factors by socio-demographic and economic characteristics during 2005–06. b. Prevalence of co-occurrence of non-communicable disease risk factors by socio-demographic and economic characteristics during 2015–16**.

**Socio-demographic and economic characteristics**	**Number of lifestyle risk factors (%)**													
	**0**		**1**		**2**		**3**		**4**			**5**		
	**Men**	**Women**	**Men**	**Women**	**Men**	**Women**	**Men**	**Women**	**Men**	**Women**		**Men**	**Women**	
Age*														
15–29	17.91	24.81	28.49	54.11	18.80	15.35	22.51	4.28	11.26	1.38		1.05	0.08	
30–49	6.72	12.61	20.77	48.26	26.15	27.23	27.19	7.74	16.67	3.92		2.50	0.25	
Residence*														
Urban	14.63	20.57	26.88	50.16	23.05	22.24	21.67	4.46	11.57	2.51		2.20	0.06	
Rural	9.90	16.14	22.53	51.94	22.70	20.93	27.29	7.78	15.95	2.93		1.62	0.28	
Education*														
No education	3.12	10.58	15.80	50.58	24.38	24.44	32.29	9.68	22.53	4.37		1.89	0.35	
Primary	5.70	14.18	19.69	49.57	23.17	24.02	30.13	8.49	18.97	3.54		2.34	0.20	
Secondary	14.34	22.30	25.46	50.72	22.45	20.72	23.80	4.36	12.12	1.82		1.84	0.08	
Higher	18.28	26.40	33.55	54.73	22.20	15.78	16.78	1.71	7.84	1.33		1.35	0.04	
Living Arrangement*														
Never married	22.94	29.81	32.69	52.05	16.82	13.21	18.57	3.55	8.18	1.28		0.79	0.10	
Living with partner	7.14	15.93	20.84	51.17	25.46	23.40	27.63	6.41	16.65	2.91		2.29	0.18	
Not living with partner	6.76	11.24	17.22	45.02	20.23	26.91	33.58	11.26	20.10	5.35		2.11	0.24	
Economic level*														
Poor	4.76	10.39	18.37	56.79	20.44	16.64	33.18	11.47	21.66	4.33		1.59	0.38	
Middle	10.41	16.22	22.20	49.98	24.15	21.87	27.07	8.44	14.51	3.29		1.66	0.21	
Rich	16.19	21.27	28.32	49.66	23.68	22.94	19.78	3.93	9.96	2.10		2.07	0.10	
Indian Region*														
North India	12.28	16.04	25.83	55.71	23.61	25.06	24.08	2.40	12.54	0.79		1.68	0.01	
Central India	7.15	16.92	19.56	55.17	22.04	18.01	28.95	6.67	20.64	3.07		1.67	0.16	
East India	6.91	15.32	19.13	53.97	18.63	19.40	30.76	7.29	21.82	3.89		2.75	0.13	
North east India	5.74	15.75	17.34	36.14	21.25	20.05	30.82	18.79	21.75	8.56		3.09	0.72	
South India	15.86	23.54	29.45	52.75	26.58	21.28	19.12	1.77	7.69	0.60		1.30	0.07	
West India	15.00	23.35	24.56	51.00	22.28	20.37	24.80	3.62	11.79	1.62		1.58	0.04	
**Total**	**12.95**	**20.07**	**24.90**	**51.41**	**23.23**	**19.30**	**22.86**	**6.49**	**13.76**	**2.57**		**2.30**	**0.16**	
**Socio-demographic and economic characteristics**	**Number of lifestyle risk factors (%)**													
	**0**		**1**		**2**		**3**		**4**		**5**		**6**	
	**Men**	**Women**	**Men**	**Women**	**Men**	**Women**	**Men**	**Women**	**Men**	**Women**	**Men**	**Women**	**Men**	**Women**
Age*														
15–29	12.19	23.11	33.87	54.04	27.48	18.03	14.14	3.56	8.96	1.14	2.72	0.12	0.65	0.00
30–49	7.75	11.71	19.54	42.67	28.04	30.89	23.03	10.27	15.31	3.66	5.92	0.75	0.41	0.05
Residence*^a^														
Urban	12.21	19.13	28.14	46.07	25.82	25.58	16.66	6.30	11.13	2.46	5.36	0.45	0.68	0.01
Rural	8.61	16.17	25.78	49.09	28.91	24.35	19.77	7.45	12.77	2.46	3.72	0.45	0.44	0.04
Education*														
No education	6.56	10.78	11.40	45.65	30.91	28.70	23.03	10.70	19.65	3.43	7.79	0.70	0.67	0.05
Primary	4.70	13.06	15.89	45.38	28.10	27.71	27.71	9.81	16.93	3.36	6.34	0.64	0.33	0.04
Secondary	10.32	19.24	29.48	49.36	26.53	23.30	18.15	5.63	10.99	2.10	3.96	0.34	0.56	0.02
Higher	15.41	25.79	35.77	50.77	30.03	19.58	9.59	2.66	7.24	1.02	1.54	0.18	0.44	0.00
Living Arrangement*														
Never married	10.19	26.24	32.64	54.81	26.54	14.47	16.50	3.20	9.94	1.12	3.84	0.15	0.35	0.01
Living with partner	9.82	15.06	23.94	46.72	28.37	27.27	19.55	7.77	13.25	2.66	4.53	0.49	0.54	0.03
Not living with partner	11.12	11.28	30.78	40.60	24.94	28.77	18.11	12.98	7.30	5.17	4.94	1.12	2.80	0.07
Economic level*														
Poor	7.26	12.81	20.71	51.92	25.81	22.69	24.13	8.83	17.08	3.11	4.37	0.58	0.65	0.06
Middle	8.77	17.60	29.66	45.53	26.67	25.76	18.21	7.75	10.98	2.83	5.47	0.51	0.24	0.02
Rich	12.63	20.19	29.77	46.41	29.82	25.84	14.54	5.44	8.93	1.79	3.74	0.32	0.57	0.01
Indian Region*														
North India	7.69	15.42	27.46	50.96	29.67	27.23	19.43	5.20	10.72	1.04	4.05	0.16	0.97	0.00
Central India	7.61	18.61	19.49	48.96	21.88	22.48	24.00	7.40	19.90	2.16	6.42	0.37	0.69	0.02
East India	7.52	14.83	20.77	54.03	30.69	24.27	19.59	5.15	16.94	1.47	3.40	0.25	1.09	0.01
North east India	5.04	14.56	15.89	33.23	20.51	22.84	29.41	17.94	20.40	9.41	7.56	1.85	1.18	0.16
South India	14.08	23.24	34.05	47.85	29.84	23.49	13.69	4.03	5.77	1.15	2.56	0.24	0.00	0.01
West India	11.10	20.72	25.31	48.97	23.76	23.65	19.23	5.19	14.08	1.33	6.52	0.14	0.00	0.01
**Total**	**8.63**	**18.76**	**25.14**	**49.30**	**25.96**	**22.39**	**21.18**	**6.84**	**13.66**	**2.28**	**4.92**	**0.40**	**0.50**	**0.03**

*Heterogeneity chi-square p value: P<0.001 for all background characteristics.

^a^Heterogeneity chi-square p value: P = 0.001 for Residence (Men).

The adjusted odds ratio on the likelihood to occurrence of multiple risk factors among Indian men and women are measured in terms of their socio-economic and demographic characteristics for the survey period of 2005–06 and 2015–16 and are shown in Table 4. It is evident that the association between simultaneous presence of multiple risk factors for both the gender in the country is higher with ageing, urban residency and among divorced or separated couples. Age shows quite consistently high and statistically significant odds for the clustering of multiple risk factors for both men and women over time. Compared the men aged 15–29, men of age 30–49 during 2005–06 have shown 36% more chances to bear two risk factors while the likelihood is observed 62% higher for the same age group of men during 2015–16. Though the odds of clustering of two risk factors among women remained the same over time the odds values show an increasing pattern by increase in number of risk factors clustering and by time. Most importantly, women of age 30–49 have shown a substantial increase in terms of the likelihood for each targeted category of clustering of multiple risk factors during the span of two survey rounds. For example, if we consider clustering of three risk factors among women, we can easily observe that the adjusted odds ratio value shows an increase from 2.4 to 3.1. It is also observed that women are more likely to show an exposure to multiple risk factors than men which remained time invariant. Though for men, place of residence shows a statistically significant association with the clustering of multiple risk factors during 2005–06 but it appears to be one of the insignificant factors for the 2015–16 time point. On the other hand, women from rural areas show a consistently lower likelihood to the clustering of multiple risk factors among them than women from the urban areas, except the case of clustering of five risk factors during 2005–06. The education pattern in clustering of risk factors is found more systematic over time among women who are secondary educated and women who are higher educated. In terms of the estimated odds values and its statistical significance, it is found that women who are secondary educated and who are higher educated are showing quite low likelihood than the no educated women for each of the clustering category of risk factors over time. Living arrangement also substantially predicts the clustering of multiple risk factors among men and women in India. During 2005–06, it is observed that men who are separated or divorced carry much higher likelihood to clustering of two, three, four and five risk factors compared the other men while this association between living arrangement of men and clustering of risk factors has shown less statistical significance in 2015–16. While living arrangement among Indian women shows quite statistically significant and consistent association with each of the types of clustering of risk factors defined in this study. In this direction, women who are divorced or separated show highest likelihood to every forms of clustering of risk factors for both the time points. The regional pattern of clustering over time shows that men and women from North-East India show higher likelihood to co-occurrence of multiple risk factors than any other regions of India over time. And women from this specific region show substantially higher likelihood to clustering of higher number of risk factors. As per economic status, clustering of multiple risk factors are found to be quite less likely among men in the rich class than those from the poor class during 2005–06 and the corresponding odds ratios are observed to be 0.85 (p-value<0.01), 0.53 (p-value<0.01), 0.56 (p-value<0.01) for two, three and four risk factors clustering respectively. On the other hand, no systematic pattern in the likelihood is observed among women for the clustering of increasing number of risk factors. Although, women from the middle class and rich class show less likelihood to clustering of three risk factors of NCDs during 2005–06 and 2015–16 respectively than the women from the poor class.

**Table 4:** Estimated adjusted odds ratio showing the likelihood to occurrence of multiple (one, two, three, four and five/six) risk factors among Indian men and women by their socio-economic and demographic characteristics, India, 2005–06.

Socio-Demographic and Economic Characteristics	Two risk factors				Three risk factors^a^				Four risk factors^a^				Five/Six risk factors^a^			
	Men		Women		Men		Women		Men		Women		Men		Women	
	2005–06	2015–16	2005–06	2015–16	2005–06	2015–16	2005–06	2015–16	2005–06	2015–16	2005–06	2015–16	2005–06^¥^	2015–16^£^	2005–06^¥^	2015–16^£^
**Age**																
15–29 ®																
30–49	1.4***	1.6***	2.0***	2.0***	1.3***	3.0***	2.4***	3.1***	1.4***	3.1***	3.4***	3.7***	1.9***	2.3***	5.4***	7.3***
**Residence**																
Urban ®																
Rural	0.9***	1.1	0.9**	0.9***	0.9***	1.1	0.9**	0.8***	0.8***	0.8	0.7***	0.6***	0.6***	0.7	1.5	0.6***
**Education**																
No education ®																
Primary	0.9**	1.1	0.9*	1	0.9*	1.2	0.8***	0.9***	0.9*	1.0	0.8***	0.8***	1.1	1.1	0.6	0.8**
Secondary	0.6***	0.7	0.8***	0.8***	0.6***	0.7	0.5***	0.5***	0.5***	0.6*	0.4***	0.6***	0.6***	0.4**	0.3***	0.5***
Higher	0.5***	0.7	0.5***	0.7***	0.4***	0.4**	0.2***	0.3***	0.3***	0.4**	0.3***	0.3***	0.3***	0.3**	0.2**	0.3***
**Living Arrangement**																
Never married ®																
Living with partner	2.1***	1.3*	1.5***	1.7***	2.3***	1.6**	1.7***	1.7***	3.1***	2.0***	1.7***	1.7***	3.9***	1.8*	1.1	1.5***
Not living with partner	2.2***	1.6	1.8***	1.9***	3.2***	1.1	2.5***	2.4***	3.8***	1.5	2.5***	2.8***	4.5***	1.8	1.0	2.6***
**Indian Region**																
North India ®																
Central India	1.3***	0.9	0.8***	0.9***	1.5***	1.2	2.2***	1.4***	1.7***	1.7*	3.1***	1.8***	1.5*	2.2*	8.4**	1.9***
East India	1.2***	1.2	0.9***	0.9***	1.8***	1.0	2.7***	0.9***	2.3***	1.6*	4.7***	1.2***	3.1***	2.1*	12.2**	1.3*
North east India	1.7***	1.4	1.4***	1.3***	2.6***	2.6***	11.5***	4.7***	3.8***	4.2***	17.1***	10.9***	6.3***	4.0***	89.6***	17.0***
South India	0.9*	0.6**	0.8***	0.8***	0.6***	0.5***	0.7***	0.7***	0.5***	0.4***	0.7*	0.9	0.6***	0.4*	7.6**	1.3*
West India	1.0	0.8	0.8***	0.8***	1.1*	1.1	1.7***	1.0	1.1	1.3	2.0***	1.1	1.2	1.1	3.4	0.8
**Economic Status**																
Poor ®																
Middle	1.00	0.9	1.4***	1.2***	0.8***	0.8	0.8***	1.0**	0.8***	0.7	0.9	1.0	1.2	1.0	0.9	0.9
Rich	0.9***	0.8	1.5***	1.2***	0.5***	0.7*	0.6***	0.8***	0.6***	0.5**	0.8**	0.8***	1.1	0.8	0.6	0.8***

^a^Adjusted for other independent variables like religion and caste.

^¥^Odds are based on five risk factors excluding hypertension as one risk factor during 2005–06.

^£^Odds are based on five/six risk factors (five and six factors were clubbed together due to low frequency) including hypertension. The 2015–16 survey data of NFHS includes hypertension as a risk factor which is not present in 2005–06 survey data.

*P* value

* p < .05;

** p < .01;

*** p < .001.

## Discussion

This study shows that clustering of multiple lifestyle risk factors of non-communicable diseases is higher amongst individuals of age 30–49 years, population having no education, among the separated and divorced and among the poor. The pattern of clustering of increasing number of non-communicable disease risk factors across gender is observed for the entire country with highest prevalence in the North-Eastern region. The NFHS, 2015–16 data reveals that, hypertension is another risk factor showing higher likelihood of co-occurrence with other risk factors of NCDs among both men and women.

Though a reduction in the prevalence has been observed for tobacco use, smoking and alcohol consumption as individual risk factor for both among men and women in India (S1 Table) clustering of two or more lifestyle risk factors has found to be widely prevalent among Indian men and women suggesting greater exposure to non-communicable diseases risk factors. Prevalence of multiple lifestyle risk behaviours are found to be higher among women in India which is in line with the study by Poortinga in 2004 [30] and suggests that aggregation of modifiable risk factors is more common among women compared to men. Consistent with the previous study findings [9], this study also confirms that persons with no formal education and who are separated carry higher chances to clustering of multiple risk factors. Studies examining the relationship between simultaneous occurrence of multiple risk factors and age reported that older individuals tend to have practiced more risk behaviours in terms of subsistence use like alcoholism and smoking [31,32] whereas another study [9] found that the prevalence of multiple risk factors is similar across ages. Increase in aggregation of lifestyle risk factors at older ages might be due to the exposure to stressful situations and the social pressure during the late stage of adolescence, where individuals become more independent in their choices [31]. Individuals from lower socioeconomic status possess lower financial resources, less education and poor access to information such as knowledge of the benefits of physical activity and healthy eating [31] and thus tend to have less healthy habits and show more clustering of risky habits. No rural-urban differential is observed in the prevalence of simultaneous presence of two or more lifestyle risk factors over time. Poorly planned work set-up especially in urban areas [33], availability of junk foods [34] and mechanization of life might be the major contributors to the increase in clustering of unfavourable lifestyle risk factors in India. Obesity has remained high in the population and has doubled amongst Indian men during the ten year period from 2005–06 to 2015–16 [28,29] which warns the need to undertake public awareness operations about the adverse effects of a fat nation. This is indicative that low intake of fruits and vegetables, sedentary behaviour and overweight are increasing among the general population in India [28,29].

A clear regional pattern has been observed where men and women residing in the North-Eastern region of the country share the highest burden of clustering of unfavourable risky lifestyle behaviours during both the survey time points than those who are living in other regions of India; while, the lowest prevalence of clustering of three or more lifestyle risk factors has been observed in the Southern region.

It has been noted that presence of one unfavourable lifestyle risk factor in turn increases the likelihood of having simultaneous presence of other risk factors demonstrating a clustering phenomenon across gender and time in India. Presence of hypertension as a lifestyle risk factor among the Indian population during 2015–16 acted as a pivot for clustering to happen given that clustering among the subjects is seen to be more prominent across both the gender during 2015–16 than 2005–06. In the current study, 13.1 percent men and approximately 7 percent women are diagnosed to suffer from high blood pressure during 2015–16. Elevated blood pressure has been recognized as one of the critical factors in developing chronic non-communicable diseases such as stroke and heart attacks [32] and therefore measures to contain increasing burden of blood pressure prevalence are the serious need of the hour. The indices of economic achievement: education and economic status indicate that clustering of risk factors is higher among the non-educated and poor population of the country which is contradictory to other developing countries where clustering is more associated among the richer people [32].

Despite presence of the synergistic effect of risk factors where clustering of unfavourable lifestyle risk behaviours are more detrimental to health, many public health intervention strategies focus on individual health behaviours in isolation. The present study is the first to describe clustering of lifestyle risk behaviours in a nationally representative population of India over two time points and across different socio-demographic and economic strata of population in India. In this study, we investigated the phenomenon of clustering of unfavourable lifestyle risk factors among Indian adults aged 15–49 years for all states and union territories of the country except Nagaland due to non-availability of the data information on the study topic. This study eventually identifies the subgroups with elevated risk to clustering of more than one risk factors and informs to build effective prevention strategies to reduce the current burden of premature mortality. The rationale behind using population aged 15–49 years for the study is firstly, information on this topic is scarce and secondly, it may provide clues for better prevention strategies to curb morbidity and mortality associated with lifestyle risk behaviours among the middle aged working population.

This study has few limitations. First, selection and defining the simultaneous presence of unfavourable risk behaviours across gender and time in the present study are inevitably subjective as the information are self-reported except the information of blood pressure from the 2015–16 round of NFHS data. Different lifestyle risk behaviours in the present study are examined by using the information based upon a self-administered questionnaire. Though the population representativeness of the survey ensures the identification of groups with unfavourable lifestyle risk factors across population of different socio-economic and demographic characteristics; assessment of unfavourable lifestyle risk behaviour like smoking, consumption of alcohol, use of smokeless tobacco in the study have been done on the basis of current use whereas for consumption of fruits, vegetables and other products has been made on the rationale of weekly, daily and no intake of mentioned food products with no specific question on the frequency of smoking, alcohol consumption and tobacco use and on the amount of food consumed by the respondent.

## Conclusion

Prevalence of unfavourable lifestyle risk behaviours associated with chronic non-communicable disease morbidity and mortality amongst Indian men and women are quite high and clustering of multiple risk factors is commonly prevalent. Both men and women have shown a substantial rise in the clustering of lifestyle risk behaviours between 2005–06 and 2015–16. As this study finds higher likelihood to co-occurrence of multiple risk factors in the middle ages in India, it is trivial to assume that India may face a significant increase in chronic non-communicable diseases in the coming decades increasing the burden to the health care services and loss of productivity due to deaths and disabilities at peak working ages. This in turn informs and recommends necessary policy implications and interventions since congregation of unfavourable lifestyle risk behaviours raises the unwanted morbidity and mortality risk by more than a summation of individual risk factors. Though the prevalence of health risk behaviour such as smoking, alcohol consumption and tobacco use has shown a decline from previous years due to the introduction of various tobacco and alcohol policies, bundling of other risk factors has increased across the population of all background characteristics especially among adult men and women belonging to 30–49 years of age. Adoption to the newly WHO recommended approach to prevent chronic disease morbidity and mortality by focusing on multiple modifiable risk factors can be a solution to prevent future premature and avoidable mortality in India due to NCDs. It is also important to promote regular physical activity, reducing sedentary behaviour and eating healthily should be emphasized and encouraged across all population subgroups.

## Supporting information

S1 Table Distribution of lifestyle risk factor among adult Indians. (DOCX)
