# Supplementary material for: Clustering of lifestyle risk factors among adult population in India: A cross-sectional analysis from 2005 to 2016
Source: PLoS One. 2021 Jan 4;16(1):e0244559. doi: 10.1371/journal.pone.0244559 (PMC7781481; doi:10.1371/journal.pone.0244559)
Supplement: S1 Table — (DOCX) [file pone.0244559.s001.docx]

**Supporting Information**

**Clustering of Lifestyle Risk Factors among Adult Population in India: A Cross-Sectional Analysis during 2005-06 and 2015-16**

**Supporting Table**

**S1 Table: Distribution of Lifestyle Risk Factor among adult Indians**

| **Risk Factor** | **Percentage (%)** | | | |  |
| --- | --- | --- | --- | --- | --- |
|  | **2005-06** | | **2015-16** | |  |
|  | **Men** | **Women** | **Men** | **Women** |  |
| Smoking | 57.46 | 11.07 | 44.49 | 9.90 |  |
| Alcohol | 34.31 | 2.64 | 30.72 | 2.35 |  |
| Smokeless tobacco | 36.70 | 9.71 | 24.01 | 7.10 |  |
| Unhealthy food | 55.09 | 58.44 | 51.36 | 61.95 |  |
| Obesity^a^ | 27.78 | 42.35 | 46.89 | 46.22 |  |
| Hypertension^b^ | na | na | 13.11 | 6.71 |  |
| ^a^ BMI>=30kg/m2 | | | | | |
| ^b^ Systolic blood pressure (>=) 140 mmHg and Diastolic blood pressure (>=) 90 mmHg | | | | | |
| na: Variable not captured during survey study | | | | | |
